# Temporal lobe dysfunction for comorbid depressive symptoms in postherpetic neuralgia patients

**DOI:** 10.1093/braincomms/fcaf132

**Published:** 2025-04-02

**Authors:** Ying Wu, Chao Wang, Wei Qian, Lieju Wang, Lina Yu, Minming Zhang, Min Yan

**Affiliations:** Department of Anesthesiology, The Second Affiliated Hospital, Zhejiang University School of Medicine, Hangzhou 310009, China; Zhejiang Key Laboratory of Pain Perception and Neuromodulation, Hangzhou 310009, China; Department of Radiology, The Second Affiliated Hospital, Zhejiang University School of Medicine, Hangzhou 310009, China; Department of Radiology, The Second Affiliated Hospital, Zhejiang University School of Medicine, Hangzhou 310009, China; Department of Anesthesiology, The Second Affiliated Hospital, Zhejiang University School of Medicine, Hangzhou 310009, China; Zhejiang Key Laboratory of Pain Perception and Neuromodulation, Hangzhou 310009, China; Department of Anesthesiology, The Second Affiliated Hospital, Zhejiang University School of Medicine, Hangzhou 310009, China; Zhejiang Key Laboratory of Pain Perception and Neuromodulation, Hangzhou 310009, China; Department of Radiology, The Second Affiliated Hospital, Zhejiang University School of Medicine, Hangzhou 310009, China; Department of Anesthesiology, The Second Affiliated Hospital, Zhejiang University School of Medicine, Hangzhou 310009, China; Zhejiang Key Laboratory of Pain Perception and Neuromodulation, Hangzhou 310009, China

**Keywords:** postherpetic neuralgia, pain-depression comorbidity, fALFF, functional connectivity, temporal lobe

## Abstract

Depression often occurs concurrently with postherpetic neuralgia (PHN), yet the neural mechanism underlying pain-depression comorbidity remains poorly understood. For this observational study, we recruited 17 depressed PHN patients, 19 non-depressed PHN patients, and 34 healthy controls (HCs) for resting-state functional MRI scans. We firstly investigated the differences in fractional amplitude of low-frequency fluctuation and regional homogeneity values among the three groups to identify a characteristic brain signal of pain-depression comorbidity. Abnormal voxel-wised functional connectivity was then compared across groups and correlated with clinical variables in each group. One-way analysis of covariance results revealed the fractional amplitude of low-frequency fluctuation values differences in the right temporal lobe (TL) and its voxel-wised connectivity with the inferior frontal gyrus (IFG) among three groups. Furthermore, the TL-IFG connectivity was positively associated with the positive emotional scores and life quality scores among depressed PHN patients, but not non-depressed PHN patients and HCs. In summary, these findings highlighted the TL dysfunction in pain-depression comorbidity among PHN population and may offer heuristic cues for central therapeutic targets that could disrupt the pain-depression vicious circle.

## Teaser

In this observational study, Wu *et al*. report the finding of temporal lobe dysfunction in pain-depression comorbidity among PHN patients by using functional MRI. This result may offer heuristic cues for central therapeutic targets that could disrupt the pain-depression vicious circle.

## Graphical Abstract

**Graphical Abstract:**
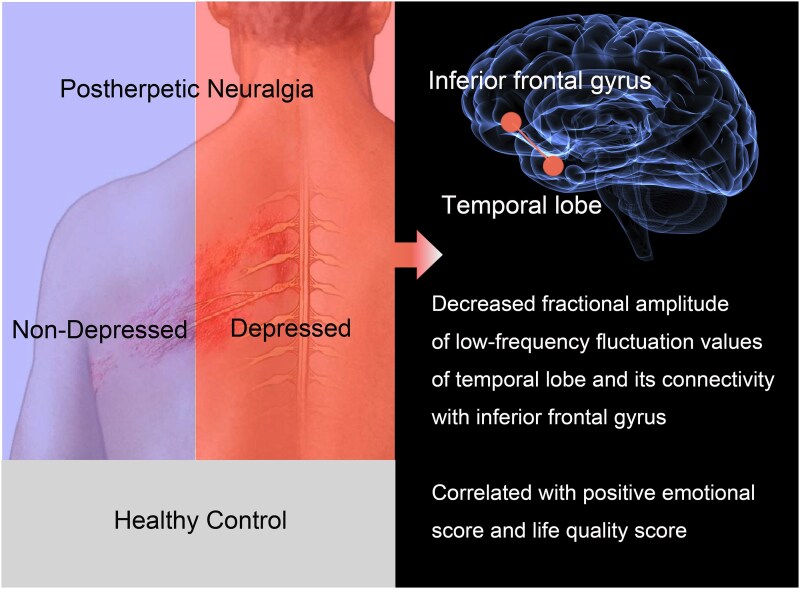

## Introduction

Chronic pain and depressive symptoms are frequently concurrent in various clinical conditions, which may complicate treatment for patients. On one hand, chronic pain, as a state of stress, is a significant risk factor for the development of depression; up to 85% of patients with chronic pain experience some degree of depressive symptoms, with increasing incidence and recurrence rates.^1-3^ On the other hand, depressive disorders can lead to somatisation symptoms of chronic pain, characterized by prolonged duration and intensity of pain, as well as pain catastrophizing.^4,5^ The population with pain-depression comorbidity may suffer from painful and psychological torture, needing more attention and exploration. Several studies have demonstrated overlaps between pain and depression with the synaptic plasticity of neural structures, neurotransmitters and pathways, which contribute to a vicious cycle of comorbid state.^6-8^ A recent human functional magnetic resonance imaging (fMRI) study revealed that compared with healthy subjects, functional connectivity (FC) of the central nucleus of amygdala (CeA)-dorsal raphe nucleus (DRN) was reduced in chronic lower back pain patients with comorbid depressive symptoms, but not in patients without depression. Furthermore, this research team also indicated a novel 5-HT^DRN^→SOM^CeA^→lateral habenula pathway in a mouse model of chronic pain by pharmacological or optogenetic approaches.^9^ Emerging rodent evidence has pointed that emotional brain regions, like insula, amygdala, anterior cingulate cortex and so on, are involved in the pathophysiology of comorbid chronic pain and depression.^10,11^ These aforementioned special overlapping regions could potentially be targeted in the treatment of chronic pain-depression comorbidity. Identifying an efficacious treatment for pain-depression comorbidity persists as a formidable challenge in recent years.^12^

Postherpetic neuralgia (PHN) stands as the most refractory complication of herpes zoster (HZ), accompanied by persist pain and comorbid depression.^13^ A 10-year prospective study demonstrated that individuals with HZ had a higher incidence of developing major depression and other depressive disorder than control group.^14^ Additionally, a multi-center study also pointed out that PHN transition was principally associated with depression.^15^ Previous studies have pointed out that the brain signal values were correlated with depressive symptom in PHN patients compared with healthy participants.^16,17^ However, the underlying brain mechanism of the pain-depression comorbidity in PHN population, which distinguish the depressed and non-depressed PHN populations, is the top priority for prevention and treatment, but still remains unclear. Therefore, in the present study, we investigated the differences of regional homogeneity (ReHo), fractional amplitude of the low frequency fluctuations (fALFF), and FC values among depressed PHN (dPHN) patients, non-depressed PHN (ndPHN) patients, and healthy controls (HCs). Our goal was to extract a distinctive brain signals associated with pain-depression comorbidity and to provide heuristic cues for potential central therapeutic targets that may disrupt the pain-depression cycle.

## Materials and methods

### Patient consent

The subjects’ consent was obtained according to the Declaration of Helsinki and it has been approved by the Ethics Committee of the Second Affiliated Hospital of Zhejiang University School of Medicine.

### Selection of study subjects

All the study procedures detailed herein received approval from the Ethics Committee of the Second Affiliated Hospital of Zhejiang University School of Medicine. Every enrollee provided written informed consent for participation. Overall, two subgroups of right-handed PHN patients (17 dPHN 19 ndPHN) and 34 HCs were recruited for study. PHN patients were diagnosed by a pain specialist in accordance with the criteria of International Association for the Study of Pain, which are as follows: (i) self-appraised pain intensity score ≥4 by Visual Analog Scale [VAS: 0 (no pain) to 10 (worst pain imaginable)]; (ii) ≥ 3-month duration after onset of acute shingles.^18^ The PHN subgroups were divided by the depression score, evaluated by the 24-item Hamilton Depression Rating Scale (HAMD) (dPHN: HAMD ≥ 8, ndPHN: HAMD < 8). Regarding the exclusion criteria, we excluded any subjects with the following conditions: (i) atypical HZ cases, such as those involving the ear, eye, viscera or asymptomatic forms; (ii) subjects currently experiencing acute or chronic pain attributable to headaches, toothaches, arthritis, and cervical or lumbar spondylopathy; (iii) any medical history of psychiatric or neurologic disorders (e.g. anxiety or depression disorder, epilepsy, or head injury) before getting shingles; (iv) any severe major health conditions, such as severe cardiovascular disorders or renal insufficiency; (v) individuals who were currently on any other medications (except for the unified medications of PHN patients); (vi) any MRI contraindications.

### Assessment of pain and emotional parameters

All questionnaires were filled out 1 h before the brain scans commenced, the same as in our previous study.^19^ Each participant filled out the short form of McGill pain questionnaire (MPQ), which consists of 11 sensory descriptors (ranging from 0 to 33) and 4 affective descriptors (ranging from 0 to 12), VAS (a total score of pain intensity by patient self-assessment, ranging from 0 to 10), present pain intensity (PPI, the present score of pain intensity before scanning, ranging from 0 to 5), ID pain score (a score assessing neuropathic pain, ranging from −1 to 5). The states of depression and anxiety were evaluated via Hamilton Depression Scale (HAMD) and Hamilton Anxiety (HAMA) Scales, ranging from 0 to 76 and 64, respectively. Participants additionally completed the two-part Positive Affect Negative Affect Schedule (PANAS), with scores ranging from 10 to 50, respectively. They also filled out the Medical Outcomes Study (MOS) 36-item short-form survey (SF-36) regarding health-related life quality, which encompasses aspects such as physical functioning, role-physical, bodily pain, general health, vitality, social functioning, role-emotional, mental health, and reported health transition (ranging from 36 to 150).

### Imaging acquisition

All MRI scans were carried out using a 3.0-Tesla MRI scanner (GE Discovery 750; GE Healthcare, Chicago, IL, USA) equipped with an 8-channel head coil in the Department of Radiology. During the MRI scanning process, each patient was placed in a supine position, with their head securely immobilized by foam pads. Additionally, earplugs were provided to mitigate noise during the scan. The patients were instructed to remain motionless for as long as possible, with their eyes closed yet staying awake. High-resolution structural T1—weighted images were obtained using a fast spoiled gradient recalled echo (GRE) pulse sequence, with the details as follows: repetition time (TR) = 7.3 ms; echo time (TE) = 3.0 ms; field of view (FOV) = 260 × 260 mm^2^; flip angle = 11°; matrix size = 256 × 256; slice thickness = 1.2 mm; totally 196 continuous sagittal slices. Resting-state fMRI (rs-fMRI) images were acquired by employing a GRE-echo planar imaging (EPI) sequence, with the following parameters: TR = 2000 ms, TE = 30 ms, flip angle = 77°, FOV = 240 × 240 mm^2^, matrix = 64 × 64, slice thickness = 4 mm, slice gap = 0 mm; 38 interleaved axial slices, scan time = 5 min 20 s.

### Rs-fMRI image preprocessing

For the preprocessing of fMRI time series volume data, the rs-fMRI Data Analysis Toolkit (REST, V1.8; http://www.restfmri.net) and Statistical Parametric Mapping (SPM12; http://www.fil.ion.ucl.ac.uk/spm) were utilized, which are based on MATLAB platform (MathWorks, Natick, MA, USA). The initial 10 volumes of each functional time series were excluded to circumvent transient signal alterations that occur prior to the magnetic field attaining a steady state. This also provided subjects with time to adapt to the scanning environment. Subsequently, the remaining images were rectified for timing disparities (with slice 37 serving as the reference) and head motion, which was determined by translation (mm) and rotation (degrees) with six parameters (three each, translation and rotation). None of the subjects were excluded due to head motion exceeding 2 mm of displacement or 2° of rotation. Subsequently, the images were spatially normalized to the Montreal Neurological Institute (MNI) space, making use of EPI templates with resampled voxel dimensions of 3 mm × 3 mm × 3 mm. Nuisance covariates were removed to prevent contamination of the reported results. These included head motion parameters, the global mean signal, white matter signal, cerebrospinal fluid signal, and other covariates such as respiratory and cardiac noise. Additionally, the linear trend in the fMRI data was eliminated.

### ReHo and fALFF analysis

The spontaneous local brain activity can be quantitatively measured by ReHo and fALFF. ReHo reflects the local coherence of spontaneous neuronal activity. For ReHo analysis, the band-pass filtering (0.01 to 0.08 Hz) was conducted to discard high-frequency physiological noise and the frequency drift <0.01 Hz.^20^ The REST 1.8 was then used to generate individual ReHo maps by calculating the Kendall's coefficient concordance (KCC) of the time series of a given voxel with those of its neighbors (26 voxels) in a voxel-wise way.^21^

The fALFF is defined as the power within the low frequency range (0.01–0.1 Hz) divided by the total power across the entire detectable frequency spectrum. As a result, it represents the relative contribution of specific low-frequency oscillations to the whole frequency range. After preprocessing, fALFF calculations were performed was described previously.^22,23^ All the generated images underwent spatial smoothing with a Gaussian kernel of 6 mm × 6 mm × 6 mm full width at half maximum (FWHM). The signal was then filtered to isolate low-frequency fluctuations within the 0.01–0.08 Hz frequency ranges, which is thought to mirror spontaneous neuronal activity. For each participant, a voxel-wise approach was utilized to extract the mean time course for fluctuations in the selected frequency range from the entire brain volume. This process enabled the creation of subject-specific fALFF maps.

### Seed-based FC analysis

Region of interests (ROI) was extracted from significantly discrepant spatial maps of ReHo or fALFF among three groups, based on the above one-way ANOVA results. Then we performed the voxel-wise FC analysis. Correlation coefficients were ultimately transformed into a normal distribution using Fisher's *z*—transformation.

### Statistical analysis

Demographic and clinical variables of dPHN, ndPHN patients and HC groups were assessed by SPSS 25.0 software. Continuous variable data are presented as mean ± standard deviation (SD). The Shapiro—Wilk test was employed to check for normality. For variables where there was no evidence against normality, differences among groups were evaluated using one-way independent analysis of variance (ANOVA) for significance. For categorical variables, comparisons among groups were made using the chi-square (χ^2^) test or Fisher's exact test. A *P*-value < 0.05 was regarded as statistically significant.

We used one-way ANCOVA analysis of variance to test the differences in ReHo, fALFF values and FC among the three groups, with the age, education level and head motion parameters as covariates. Significance was set at the voxel level threshold of *P* < 0.001 followed by cluster-wise correction of *P* < 0.05. Post-hoc analysis with the least significant difference method was performed to further identify where the difference came from.

Finally, to investigate the correlations between significant brain values (ReHo, fALFF and FC) and clinical variables (duration, MPQ, VAS, PPI, ID pain, HAMD, HAMA, PANAS, SF-36 scores), partial correlation analyses were performed. These analyses were carried out to eliminate the influence of differences in age, gender, and years of education. Statistical significance was set at *P* < 0.05, with false discovery rate (FDR) correction applied.

## Results

### Demographic and clinical characteristics

In the present study, a total of 70 subjects were ultimately included, including 17 dPHN patients, 19 ndPHN patients, and 34 HCs. The demographic and clinical characteristics of the participants are presented in Table 1. There was a difference in age (years, mean ± SD: dPHN: 63.53 ± 8.54; ndPHN: 67.32 ± 6.72; HC: 58.44 ± 7.94). There were no statistically significant differences in terms of educational level (years, mean ± SD: dPHN: 7.52 ± 3.68; ndPHN: 7.76 ± 4.11; HC: 6.06 ± 4.31) and gender (males/females, dPHN: 10/7; ndPHN: 16/3; HC: 18/17). In the subsequent analysis, we used the age, educational level and gender as covariates.

**Table 1:** Demographic and clinical variables of dPHN, ndPHN patients and HCs

	HC (*n* = 34)	dPHN (*n* = 17)	ndPHN (*n* = 19)	*F*	*P*-value
Demographic data					
Age (years, mean ± SD)	58.44 ± 7.94 ^b^	63.53 ± 8.54	67.32 ± 6.72	8.310	0.001*
Gender (males/females)	18/17	10/7	16/3	5.710	0.058
Education (years, mean ± SD)	6.06 ± 4.31	7.52 ± 3.68	7.76 ± 4.11	1.329	0.272
Duration from first onset (months, mean ± SD)	n.a.	18.44 ± 22.49	12.18 ± 14.38	n.a.	n.a.
Clinical data					
Laterality (Left/Right)	n.a.	9/8	10/9	n.a.	n.a.
Rash location					
Cervical	n.a.	4	1	n.a.	n.a.
Thoracic	n.a.	12	17	n.a.	n.a.
Lumbar	n.a.	0	1	n.a.	n.a.
Sacral	n.a.	1	0	n.a.	n.a.
Pain VAS (0–10) (mean ± SD)	n.a.	5.82 ± 1.13	5.0 ± 0.88 ^c^	n.a.	n.a.
MPQ sensory (mean ± SD)	n.a.	9.00 ± 2.94	5.95 ± 2.15 ^c^	n.a.	n.a.
MPQ affective (mean ± SD)	n.a.	2.71 ± 1.11	1.58 ± 0.77 ^c^	n.a.	n.a.
PPI (mean ± SD)	n.a.	2.82 ± 0.39	2.26 ± 0.56 ^c^	n.a.	n.a.
ID pain (mean ± SD)	n.a.	2.71 ± 1.26	2.11 ± 0.81	n.a.	n.a.
Psychological data					
HAMD (mean ± SD)	1.26 ± 1.58^a,b^	9.59 ± 1.46	4.42 ± 1.39^c^	174.221	<0.001*
HAMA (mean ± SD)	0.68 ± 1.17^a,b^	6.06 ± 2.01	2.79 ± 1.35^c^	76.954	<0.001*
PANAS positive (mean ± SD)	24.12 ± 2.31^a,b^	12.59 ± 1.66	12.32 ± 1.8	287.456	<0.001*
PANAS negative (mean ± SD)	10.62 ± 1.18 ^a,b^	18.53 ± 2.67	14.73 ± 2.51^c^	90.470	<0.001*
SF-36 (mean ± SD)	136.08 ± 3.35^a,b^	108.58 ± 7.50	116.82 ± 5.69^c^	180.537	<0.001*

^a^Significant difference between HC and dPHN.

^b^Significant difference between HC and ndPHN.

^c^Significant difference between dPHN and ndPHN.

dPHN, depressed postherpetic neuralgia; HAMA, Hamilton Anxiety Scale; HAMD, Hamilton Depression Scale; HC, healthy controls; MPQ, McGill pain questionnaire; n.a., not available; ndPHN, non-depressed postherpetic neuralgia; PANAS, Positive Affect Negative Affect Score; PPI, present pain intensity; SF-36, 36-item short form from health survey; VAS, Visual Analogous Scale.

Compared with HCs, ndPHN and dPHN patients showed higher depressive, anxious scores and PANAS negative score, lower PANAS positive score and life quality score. The dPHN group showed significantly higher pain scores (MPQ sensory and affective, VAS, PPI score), higher depressive, anxious scores and PANAS negative scores, lower PANAS positive score and life quality score compared with ndPHN patients (Table 1).

### Comparison of ReHo and fALFF between dPHN, ndPHN patients, and HCs

**Figure 1:**
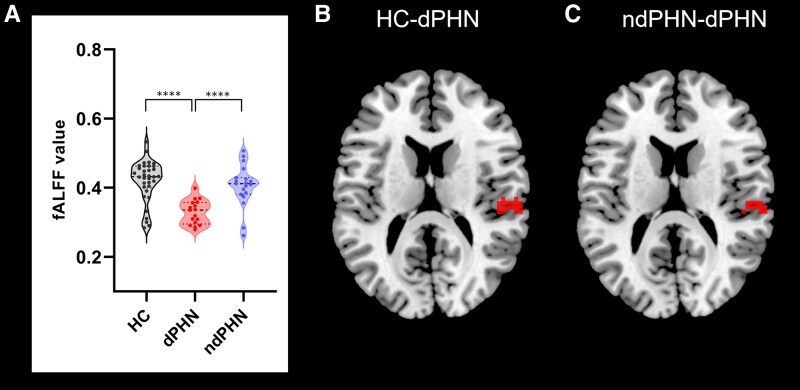
**Comparison of fALFF values between dPHN (*N* = 17), ndPHN patients (*N* = 19), and HCs (*N* = 34).** (**A**) The significant differences of fALFF values between three groups (ANCOVA, *F* = 16.02, voxel *P*-value < 0.0001, cluster *P*-value < 0.05, GRF-corrected). (**B**) Comparison of fALFF values between dPHN patients and HCs (*t*-test, *t* = 5.81, *P* < 0.0001). Highlighted regions represent the fALFF value difference of right TL between dPHN patients and HCs. (**C**) Comparison of fALFF values between dPHN and ndPHN patients (*t*-test, *t* = 4.52, *P* < 0.0001). Red regions represent the fALFF value difference of right TL between dPHN and ndPHN patients. ANCOVA, analysis of covariance; dPHN, depressed postherpetic neuralgia; fALFF, fractional amplitude of the low frequency fluctuations; GRF, Gaussian Random Field; HC, healthy control; ndPHN, non-depressed postherpetic neuralgia; TL, temporal lobe. Dots represent the fALFF value of each subject.

One-way ANCOVA results showed the differences of fALFF values among three groups (voxel *P*-value < 0.0001, cluster *P*-value < 0.05, GRF-corrected) (Fig. 1A, Table 2). The dPHN group significantly showed lower fALFF values in right temporal lobe (TL) compared with ndPHN group and HCs (Fig. 1B, *P* < 0.0001; Fig. 1C, *P* < 0.0001). There were no significant differences in KCC-ReHo value among the three groups.

**Table 2:** Clusters of different fALFF between dPHN, ndPHN patients and HCs (voxel *P*-value < 0.001, cluster *P*-value < 0.05, GRF-corrected)

Brain regions	L/R/B	Cluster size	Peak MNI coordinate			Peak *T* value
			X	Y	Z	
Temporal lobe	R	34	63	−24	12	4.376

B, Bilateral; dPHN, depressed postherpetic neuralgia; GRF, Gaussian random field; HC, healthy controls; L, Left; MNI, Montreal Neurological Institute; ndPHN, non-depressed postherpetic neuralgia; R, Right; X, Y, Z, coordinates of primary peak locations in the MNI space.

### Comparison of TL voxel-wised connectivity between dPHN, ndPHN patients and HCs

**Figure 2:**
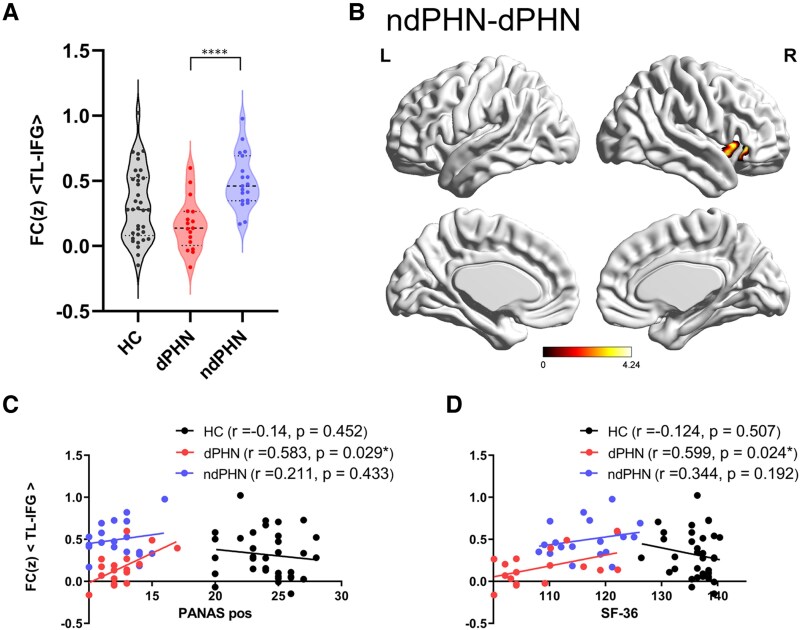
**Differences of TL voxel-wised connectivity between groups and correlation patterns with clinical symptoms.** (**A**) The significant differences of TL voxel-wised connectivity between dPHN (*N* = 17), ndPHN patients (*N* = 19), and HCs (*N* = 34) (ANCOVA, F = 8.511, *P* < 0.001, Alphasim-corrected, cluster size >22). (**B**) Visualisation of TL voxel-wised connectivity difference between dPHN and ndPHN patients. L, left; R, right. Coloured regions represent the difference of right IFG, which connect to right TL. (**C**) Correlation between TL-IFG connectivity and Positive-PANAS score (Partial correlation analysis. HC, *N* = 34, *r* = −0.14, *P* = 0.452; dPHN, *N* = 17, *r* = 0.583, *P* = 0.029; ndPHN, *N* = 19, *r* = 0.211, *P* = 0.433). (**D**) Correlation between TL-IFG connectivity and SF-36 score (Partial correlation analysis. HC, *N* = 34, *r* = −0.124, *P* = 0.507; dPHN, *N* = 17, *r* = 0.599, *P* = 0.024; ndPHN, *N* = 19, *r* = 0.344, *P* = 0.192). ANCOVA, analysis of covariance; dPHN, depressed postherpetic neuralgia; HC, healthy control; IFG, inferior frontal gyrus; ndPHN, non-depressed postherpetic neuralgia; PANAS, positive affect negative affect schedule; TL, temporal lobe; SF-36, the MOS 36-item short-form survey. Dots represent the TL-IFG connectivity value of each subject.

One-way ANCOVA results showed significant differences in right TL voxel-wised connectivity with inferior frontal gyrus (IFG) among three groups (Alphasim correction of *P* < 0.001, cluster size >22) (Fig. 2A and B, Table 3). The dPHN group significantly showed lower TL-IFG connectivity compared with ndPHN group (Fig. 2B, *P* < 0.0001). There were no significant differences in TL-IFG connectivity between HCs and other two groups.

**Table 3:** Clusters of different TL voxel-wised connectivity between dPHN, ndPHN patients and HCs (alphasim correction of *P* < 0.01, cluster size >22)

Brain regions	L/R/B	Cluster size	Peak MNI coordinate			Peak *T* value
			X	Y	Z	
Inferior frontal gyrus	R	69	36	33	−6	10.096

B, Bilateral; dPHN, depressed postherpetic neuralgia; HC, healthy controls; L, Left; MNI, Montreal Neurological Institute; ndPHN, non-depressed postherpetic neuralgia; R, Right; TL, temporal lobe; X, Y, Z, coordinates of primary peak locations in the MNI space.

### Correlation patterns of brain values with clinical symptoms

By using the partial correlation analysis between abnormal fALFF values or voxel-wised connectivities and clinical symptoms, we discovered that right TL-IFG connectivity was positively associated with the PANAS positive score (Fig. 2C, *r*= 0.583, *P* = 0.029) and SF-36 (Fig. 2D, *r* = 0.599, *P* = 0.024) in dPHN patients, while there was no correlation in ndPHN group and HCs (FDR corrected). No correlations were found in TL fALFF values and clinical scores in three groups (Supplemental Table 1).

## Discussion

To the best of our knowledge, this is the first to investigate the neural mechanism underlying pain-depression comorbidity in PHN patients. A summary of the key findings is depicted here and emphasized as follows: (i) The dPHN group significantly exhibited lower fALFF value in right TL compared with both ndPHN group and HCs; (ii) The dPHN group significantly showed lower right TL-IFG connectivity compared with ndPHN group; (iii) The TL-IFG connectivity exhibited a positive association with the PANAS positive score and SF-36 in dPHN patients, while there was no correlation in ndPHN group and HCs.

### TL and pain

TL is one brain area usually responsible for memory and cognition; however, several researches have indicated that it’s also involved in pain perception and modulation in human neuropathic states and in animal models.^24^ Neuroimaging studies of healthy subjects have shown activation of various medial TL regions in response to acute nociceptive stimuli in various modalities and in different tissue types, including cutaneous, muscular, and visceral.^25-33^ In addition to the above studies in health subjects, aberrant activity of medial TL was also found in several chronic pain stages, including post-surgical pain, chronic back pain, migraine, myofascial pain syndrome, primary trigeminal neuralgia and so on.^34-39^ Reduced medial TL-hippocampus FC and reduced medial TL volume can also act as neuroimaging biomarkers to predict the transition from sub-acute to chronic pain, which indicated that TL function in the early phase may be associated with the chronic pain prognosis.^34,40,41^ Interestingly, the reduced deactivation of medial TL in fibromyalgia patients in the ensuing post stimulation period, called painful after-sensations, exhibited its association with clinical pain and catastrophizing.^42^ Similarly, the PHN group in our study (dPHN plus ndPHN group) significantly showed lower TL fALFF value compared with HCs and was correlated with the emotional scores and life quality score (shown in Supplemental Fig. 1). This was in accordance with the previous perspective,^16,17,43^ indicating that decreased TL activity may serve as a potential biomarker in the chronic pain condition of HZ patients.

### TL and depression

Temporal brain dysfunction has been indicated to contribute to the neurobiological mechanism of the depression symptoms of depression.^44^

The MTL is part of TL regions that has been studied the most, initially acts as the default mode network subsystems, which plays a pivotal role in the emotion processing, detection of surrounding environment, the inner mind activities such as meditation, introspection, and envisioning future.^45^ The MTL subsystem contributes to rumination and its sub-component process in major depressive disorder. This is due to its inherent connection with the psychological processes of self-referential processing and autobiographical memory.^46,47^ A meta-analysis revealed that disrupted resting-state FC of the middle temporal gyrus (MTG)—posterior cingulate along with the deviated grey matter of left MTG, have been highlighted to subserve cognitive control and affective regulation in major depressive disorder patients.^48^ In a longitudinal population of the elderly with 5-year follow-up, the presence of TL atrophy and moderate-to-severe white matter lesions independently predicted major depression, suggesting that both neurodegeneration and cerebrovascular disease should be related to preventable factors.^49,50^ These researches hint that presence of TL dysfunction is related to not only the current depression state but also the latter permanent depressive outcome. Our research findings suggested that the dPHN population exhibited lower right TL fALFF values and TL-IFG connectivity compared with ndHN patients, which was consistent with previous viewpoint. These indicated that the decreased TL activity may contribute to the depressive state of PHN patients and may be a therapeutic target for PHN patients with comorbid depression.

### TL and pain comorbidities

Pain-depression comorbidity has high prevalence in the chronic pain population, which has a significant socioeconomic impact on society. With the bidirectional and the underlying neurobiology ‘shared’ between pain and depression, the identification of an efficacious treatment target for the pain-depression comorbidity poses a significant challenge.^51^ Previous studies have shown that the abnormal mPFC has been proposed to be a therapeutic target for migraineurs with comorbid depression, which may contribute to determining the common symptoms in migraine with comorbid depression.^52^ The practice of bilateral anterior cingulotomy was substantiated to effectively relieve symptoms associated with comorbid neuropathic pain and MDD.^53^ The latest and comprehensive investigation of pain-depression comorbidity in a chronic pain animal model has specifically unveiled a neural circuit encompassing the dorsal raphe nucleus and amygdala. Moreover, this study has also corroborated the association between amygdala connectivity and pain-depression comorbidity in human studies.^9^ The TL has also been pointed out in both pain and depression researches independently,^38,50^ meanwhile the grey matter volume and regional cerebral blood flow of TL have been found in fibromyalgia patients and burning mouth syndrome patients, with its association with the severity of depression.^54,55^ However, the precise association between TL and emotional state in chronic pain with or without depression subpopulations remains uncertain. Thus our present study filled some of the gaps that identified the role of TL activity and its connectivity in depressed PHN patients compared with ndPHN.

In addition, pain comorbidities may also be involved in the cognitive function. In most patients experiencing pain, it is challenging to establish the temporal relationship among pain, cognitive deficits, anxiety, and depression. Longitudinal animal studies suggested that emotional and cognitive alterations may, in some cases, begin long after the onset of pain.^56,57^ Many brain regions may be involving in the cognitive dimensions of pain processing, TL is also included.^58^ An intriguing convergence of nociception and cognition arises from a case report, patient H.M. whose bilateral medial temporal lobe (MTL) was resected for epilepsy, exhibited heightened thermal pain tolerance with global amnesia.^59^ Recent similar studies have also shown that non-hispanic black participants without dementia but with high chronic knee pain stage appeared to have thinner temporal cortex in areas associated with Alzheimer’s disease.^60^ Numerous longitudinal studies have likewise discovered that older adults presenting with depressive symptoms of depression are at an elevated risk of cognitive impairment.^61,62^ Thus, there appear to be complex relationships between TL, chronic pain stage, cognition disease, emotional state and sociodemographic variables, more exploration is needed.

## Limitations

Despite our first findings on the neural mechanism of pain-depression comorbidity in PHN patients, this study still has limitations. Firstly, given the limited availability of adequate data on patients with PHN, this inquiry will be addressed through prospective imaging studies encompassing a larger sample size. Secondly, we did not include the acute HZ population and clarify the relationship between depression degree along with brain values in acute period and final HZ outcome. If we were to conduct future studies, the predictive value of these acute parameters could be utilized to guide the administration of combination antidepressant therapy in order to mitigate the transition rate of PHN. Thirdly, the ROIs were extracted from significantly discrepant spatial maps of ReHo or fALFF, which may introduce a potential concern about circularity and bias the FC results towards significance.^63,64^ We would pay more attention and advance the analysis in the further study. Finally, due to the almost significant difference in terms of gender in our study, though there were no differences in the emotional scores between the male and female group, the complex relationships between pain-depression comorbidity and sex dimorphism need further investigation and would be controlled more homogeneous.

## Conclusion

In this study, the dPHN group significantly exhibited lower right TL fALFF values and TL-IFG connectivity compared with ndPHN and exhibited a positive association with the clinical emotional scores. These results identified the abnormal TL function in pain-depression comorbidity of PHN population and may provide heuristic insights for central therapeutic targets that may interrupt the pain-depression vicious circle.

## Supplementary Material

fcaf132_Supplementary_Data

## Data Availability

The data that support the findings of this study are available on request from the corresponding author.
