# Supplementary material for: Temporal lobe dysfunction for comorbid depressive symptoms in postherpetic neuralgia patients
Source: Brain Commun. 2025 Apr 2;7(2):fcaf132. doi: 10.1093/braincomms/fcaf132 (PMC11985680; doi:10.1093/braincomms/fcaf132)
Supplement: fcaf132_Supplementary_Data [file fcaf132_supplementary_data.pdf]

Supplementary Figure 1. Comparison of TL fALFF values between PHN patients(N=36) and HCs (N=34), with its correlation with clinical symptoms.

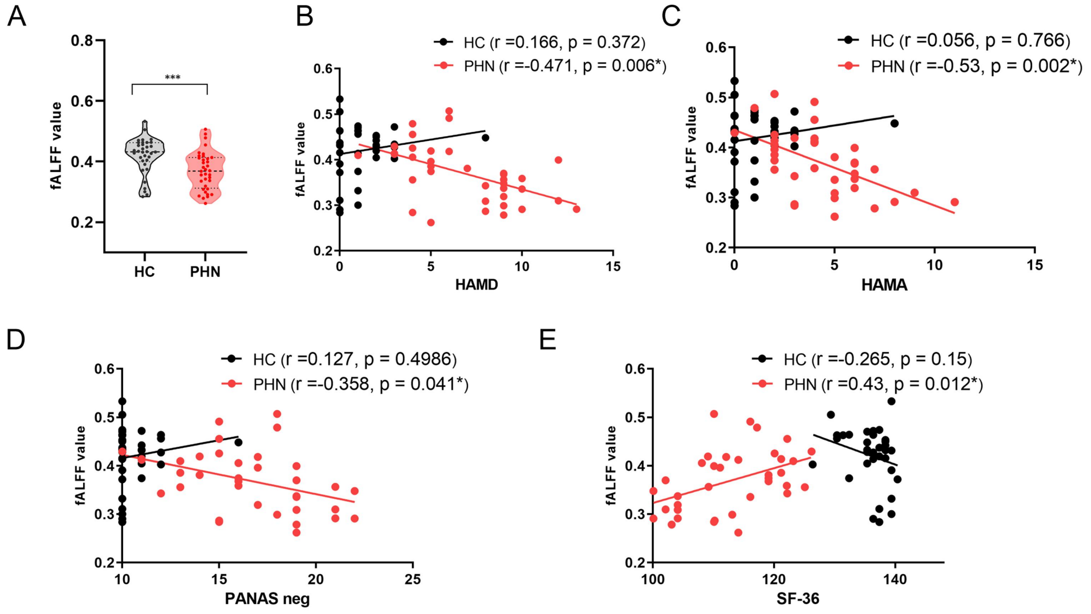

A. Comparison of TL fALFF value between PHN patients and HCs (t-test, t=3.47, P = 0.0009).

B. Correlation between TL fALFF value and HAMD score (Partial correlation analysis. HC, N=34, r= 0.166, *p*=0.372; PHN, N=36, r= -0.471, *p*=0.006).

C. Correlation between TL fALFF value and HAMA score (Partial correlation analysis. HC, N=34, r= 0.056, *p*=0.766; PHN, N=36, r= -0.53, *p*=0.002).

D. Correlation between TL fALFF value and Negative-PANAS score (Partial correlation analysis. HC, N=34, r= 0.127, *p*=0.4986; PHN, N=36, r= -0.358, *p*=0.041).

E. Correlation between TL fALFF value and SF-36 score (Partial correlation analysis. HC, N=34, r= -0.265, *p*=0.15; PHN, N=36, r= 0.43, *p*=0.012).

fALFF, fractional amplitude of the low frequency fluctuations; PHN, postherpetic neuralgia; HC, healthy control; TL, temporal lobe; HAMD, Hamilton Depression Scale; HAMA, Hamilton Anxiety Scale; PANAS, Positive Affect Negative Affect Schedule; SF-36, the Medical Outcomes Study (MOS) 36-item short-form survey.

Dots represent the fALFF value of each subject. Black, HC; red, PHN.

Supplemental Table 1. Correlation between TL fALFF values and clinical symptoms in dPHN, ndPHN patients and HCs.

r	HC (n=34)	dPHN (n=17)	ndPHN (n=19)
Duration	NA	-0.191	0.194
MPQ sensory	0.094	0.154	-0.079
MPQ affective	NA	0.236	0.009
VAS	NA	0.142	-0.07
PPI	NA	0.149	0.15
ID PAIN	NA	0.046	0.083
HAMD	0.166	0.219	-0.115
HAMA	0.056	-0.171	-0.494
PANAS pos	-0.267	-0.19	-0.186
PANAS neg	0.127	-0.113	-0.012
SF-36	-0.265	0.298	0.191

NA: Not Available; dPHN, depressed postherpetic neuralgia; ndPHN, non-depressed postherpetic neuralgia; HC, healthy control; TL, temporal lobe; VAS, Visual Analogous Scale; MPQ, McGill pain questionnaire; PPI, present pain intensity; HAMD, Hamilton Depression Scale; HAMA, Hamilton Anxiety Scale; PANAS, Positive Affect Negative Affect Score; SF-36, 36-item short form from health survey.
